# Effects of short- and long-term TSH suppression on lumbar bone mineral density in both genders using PET/CT

**DOI:** 10.1038/s41598-023-50118-z

**Published:** 2023-12-19

**Authors:** Holger Einspieler, Christoph Walter, Marcus Hacker, Georgios Karanikas, Dietmar Tamandl

**Affiliations:** 1 Division of Nuclear Medicine, Department of Biomedical Imaging and Image-Guided Therapy, Medical University of Vienna, Vienna, Austria; 2 Division of General and Pediatric Radiology, Department of Biomedical Imaging and Image-Guided Therapy, Medical University of Vienna, Vienna, Austria

**Keywords:** Endocrine system and metabolic diseases, Endocrinology, Oncology, Risk factors

## Abstract

Iatrogenic subclinical hyperthyroidism is induced intentionally in patients with differentiated thyroid cancer to reduce the risk of tumor recurrence. This retrospective study aimed to investigate the effect of thyroid-stimulating hormone (TSH) suppressive therapy on bone mineral density in men and women. Two cohorts of endocrine cancer patients were compared. In cohort A, 42 patients with long-lasting suppressed serum TSH were assessed. Cohort B consisted of 41 euthyroid patients. Bone density was measured in the L1-L4 lumbar vertebrae of all patients using PET/CT scans performed for cancer staging. In 17 patients of cohort A who received a second PET/CT scan, bone density was measured again to provide longitudinal analysis. A non-significant difference in age (*p* = .572) and equal distribution of sex (*p* = .916) was determined when comparing both cohorts. A significant difference (p = .011) with a moderate effect (η^2^ = .08; 20.4%) was observed regarding higher bone mineral density (BMD^HU) in cohort B with normal TSH levels (M 160.63 ± 54.7 HU) versus cohort A under TSH suppression therapy (M 127.9 ± 59.5 HU) for a mean duration of 4.45 ± 2.64 years. Furthermore, no significant change in BMD^HU (*p* = .786) was found in those patients who received a second PET/CT scan after a mean observation time of 2.3 ± 1.2 years. In conclusion, long-lasting TSH suppression therapy caused a statistically significant decrease in BMD^HU while short-lasting therapy didn't. Therefore, we can assume a higher likelihood of osteoporosis in those patients under prolonged TSH suppression.

## Introduction

Subclinical hyperthyroidism is defined by a serum thyroid-stimulating hormone (TSH) level below the reference range, as well as normal free thyroxine (T4) and free triiodothyronine (T3) concentrations^1^. The prevalence of subclinical hyperthyroidism increases with age and occurs more often in women, with an estimated incidence of 0.7% to 12.4%. This wide span in percentage is the result of multiple factors, such as varying diagnostic criteria, measurement methods for TSH, and iodine intake^2–4^.

Subclinical hyperthyroidism is generally associated with several clinical conditions, such as atrial fibrillation, cardiac dysfunction, and osteoporosis^5–7^, and can be caused endogenously and exogenously. The most common causes for endogenous subclinical hyperthyroidism include Graves' disease, toxic adenoma, and toxic multinodular goiter^8,9^. Graves' disease is more frequent in patients younger than 65 years old in iodine-replete areas, compared to toxic adenoma and toxic multinodular goiter, which are relatively more common causes in iodine-deficient areas and in elderly people (≥ 65 years)^10^. Exogenous causes include unintentional thyroid hormone overtreatment in patients with hypothyroidism, purposeful overuse by patients (often surreptitious), as well as the intentional suppression of TSH in thyroid carcinoma patients^8,11^. Thyroid cancer is one of the most common cancers worldwide, with a high survival rate due to early diagnosis and improved treatments, especially in differentiated thyroid cancer (DTC)^12^. In patients suffering from DTC, suppressed serum TSH values are intentionally induced after primary treatment (total thyroidectomy and, if applicable, radioiodine ablation)^13^. Ultimately, suppressed TSH concentration, defined as a TSH value below 0.1mU/liter^14^, leads to a reduced risk of tumor recurrence^13^.

Osteoporosis, the most common chronic metabolic bone disease worldwide, most frequently occurs in the elderly and especially in women^15^. It is characterized by low bone mass, decline of bone tissue, and disruption of bone microarchitecture, which finally can result in a reduction of bone strength and an increased risk of fractures^16^.

Bone mineral density (BMD) testing correlates with bone strength and therefore represents an excellent predictor of future fracture risk^17^. Although dual-energy x-ray absorptiometry (DXA) is considered the gold standard for the measurement of BMD, mainly due to the relatively minimal radiation exposure, Hounsfield unit (HU) measurements obtained from CT scans can predict BMD as well^18–20^.

There are several risk factors involved with osteoporosis, such as estrogen deficiency, Vitamin D insufficiency, and hyperparathyroidism^21–24^. In addition, it is well known that clinical hyperthyroidism induces high bone turnover, which can lead to osteoporosis, and therefore, increased fragility fractures^25^. Thyroid hormones, especially T3, affects the bone remodeling cycle, and therefore, thyroid dysfunction can result in increased bone turnover^26^.

Interestingly, the effect of subclinical hyperthyroidism on osteoporosis is still unclear. Although studies reported TSH receptor expression on the surface of bone cells^27,28^, the extent of the contribution to bone loss by suppressive TSH levels remains controversial.

On the one hand, some studies indicate that suppressed TSH levels cause lower BMD^29–31^. On the other hand, various studies demonstrate the opposite and report that there is no significant difference regarding BMD in those patients^32–35^. Primarily due to these controversies and because of the clinical importance, we decided to examine bone densities in two age- and sex-matched cohorts with significantly different TSH values over a long period of time. Compared to the other studies previously mentioned, we used CT to determine BMD^HU.

Second, to provide longitudinal analyses and validate other studies with shorter intervals of TSH suppression^32,36^, we also measured BMD^HU within those patients who received a second PET/CT scan.

## Methods

This study was designed as a retrospective data analysis, comparing two cohorts with different serum TSH values in patients. AKIM (Allgemeines Krankenhaus Informations Management) was used to extract patients’ data. All blood examinations were performed in the laboratory at the Vienna General Hospital (AKH). Cohort A consisted of patients who were treated for DTC in the period from 01.01.2016–31.12.2020 in the Department of Nuclear Medicine at the Vienna General Hospital. In cohort B, patients were included who were treated for endocrine cancer from 01.01.2020–31.03.2022 in the Department of Nuclear Medicine at the Vienna General Hospital. Ethical approval was obtained by the medical ethics committee of the Medical University of Vienna (EK-No. 1958/2021 of November 9th, 2021) and the need for informed consent was waived by the committee. We confirm that all research was performed in accordance with relevant guidelines and regulations.

### Cohort A

Patients belonging to cohort A were treated for a DTC. They received surgery and radioiodine therapy. Furthermore, these patients were supplemented with levothyroxine to reach TSH suppression. In cohort A, 116 patients met the inclusion criteria. Patients were required to be under TSH suppression therapy for at least six months, to be at least 18 years old, and to have undergone at least one 18F-FDG PET/CT during the observation period.

For cohort A, 74 patients were excluded for the following reasons: TSH not degraded/suppressed (< 0.40mU/l); TSH suppression therapy shorter than six months; no data available in AKIM; previous illnesses (diabetes mellitus types 1 and 2, calcium metabolism disorder, severe vitamin D deficiency, osteoporosis, bone neoplasms); cortisone medication; and increased fT3 and/or fT4 (above the reference values).

### Cohort B

Patients belonging to cohort B were treated for endocrinological cancer and did not receive TSH suppression therapy. They had to be at least 18 years old and had to have undergone at least one F18 DOPA PET/CT during the observation period. 94 patients met the inclusion criteria.

For cohort B, 53 patients were excluded for the following reasons: TSH degraded/suppressed (< 0,40mU/l); TSH suppression therapy; no data available in AKIM; previous illnesses (diabetes mellitus types 1 and 2, calcium metabolism disorder, severe vitamin d deficiency, osteoporosis, bone neoplasms); cortisone medication; and increased fT3 and/or fT4 (above the reference values).

The remaining patients suffered from different endocrinological diseases: pheochromocytoma (n = 14); medullary thyroid cancer (n = 10); paraganglioma (n = 5); adrenal tumor (n = 4); insulinoma (n = 4); multiple endocrine neoplasia (n = 2); glomus tumor (n = 1); and bronchial carcinoma (n = 1).

### PET/CT procedures

Patients in both cohorts underwent at least one PET/CT scan. Bone density was measured in the L1-L4 lumbar vertebrae of all patients using PET/CT scans performed for initial staging, follow-up, or restaging. A multi-slice computed tomography (CT) in axial orientation and a positron emission tomography (PET) were performed. Of 83 patients, 64 underwent an intravenous contrast-enhanced CT study. [18F]-FDG was applied in patients of cohort A, and [18F]- DOPA in patients of cohort B, respectively.

### Image analysis

The PET/CT images were analyzed using Agfa HealthCare`s IMPAX EE by one reader. The axial section plane of the whole-body bone sequence was used to set the region of interest (ROI) in the L1 to L4 vertebral bodies, after angulating parallel to the respective end plates. After setting the largest possible ROI and thereby leaving out the hyperdense edges of the vertebral bodies, the average Hounsfield Unit value of the ROI were measured, referred to as mean Hounsfield Units (BMD^HU m) and the corresponding standard deviation of the mineral content of the ROI, referred to as standard deviation HU (BMD^HU sd). HU values were measured as in previously published methods^37–39^. Patients with signs of fractures or tumor manifestations in the L1 to L4 vertebral bodies were excluded from analysis.

### Statistical analysis

#### Descriptive statistics

Key values such as mean (M), standard deviation (SD), minimum (min), maximum (max), as well as median (Md), and the corresponding measure of dispersion interquartile range (IQR), were used for characterizing metric parameters. Boxplots and a scatterplot were generated.

#### Inferential statistics

The significance level was set at α = 5%, corresponding to the probability of a type I error, so that p-values of 0.05 or less was considered statistically significant. All statistical analyses were performed using IBM SPSS® version 27.0.1 (IBM, Armonk, NY).

According to Cohen’s classification, ranges of the effect size *r* ≥ 0.10 are described as small, r ≥ 0.30 as moderate, and *r* ≥ 0.50 as large. The expression *r* = z / √N was used for calculation. Regarding the effect size η^2^ (eta-square), ranges ≥ 0.01 are described as small, ≥ 0.06 as medium, and ≥ 0.14 as large.

Nominally scaled variables including frequencies (n) and associated percentages (%) were calculated as well. The corresponding confidence interval [CI- 95%: LL; UL] for the location of the expected value of the probability was determined if necessary, using the expression π_1,2_ = *p* ± z_α/2_ √(p*(1-p))/n. According, the corresponding two-tailed z-value of 1.96 was used for the 5% error probability of the CI.

To assess the proportion of female and male patients within both cohorts, Chi-square testing based on cross tabulation was used.

The non-parametric Mann–Whitney U-test was used to evaluate differences in age, TSH values, BMI, and further laboratory parameters within both cohorts as well as to compare BMD^HU m regarding patients` sex. A paired t-test was performed to examine a change in BMD^HU m in L1-L4 within cohort A between the two PET/CT scans.

The relation between two metric parameters was assessed by bivariate scatterplots, considering the adequate regression function. To demonstrate relationships between two interval-scaled variables, the Pearson’s coefficient was used.

To account for patient sex in addition to cohorts A and B in BMD^HU, the difference in BMD^HU m was assessed using two-way (2 × 2) ANOVA. Differences between both cohorts regarding the BMD^HU m from the L1 to L4 vertebrae were conducted using one-way, multivariate analysis of variance (MANOVA).

## Results

The patient collective consisted of 83 patients and was classified into two groups based on their TSH values: Patients with DTC and simultaneously suppressed TSH were assigned to cohort A, patients with normal TSH values were included in cohort B.

**Figure 1:**
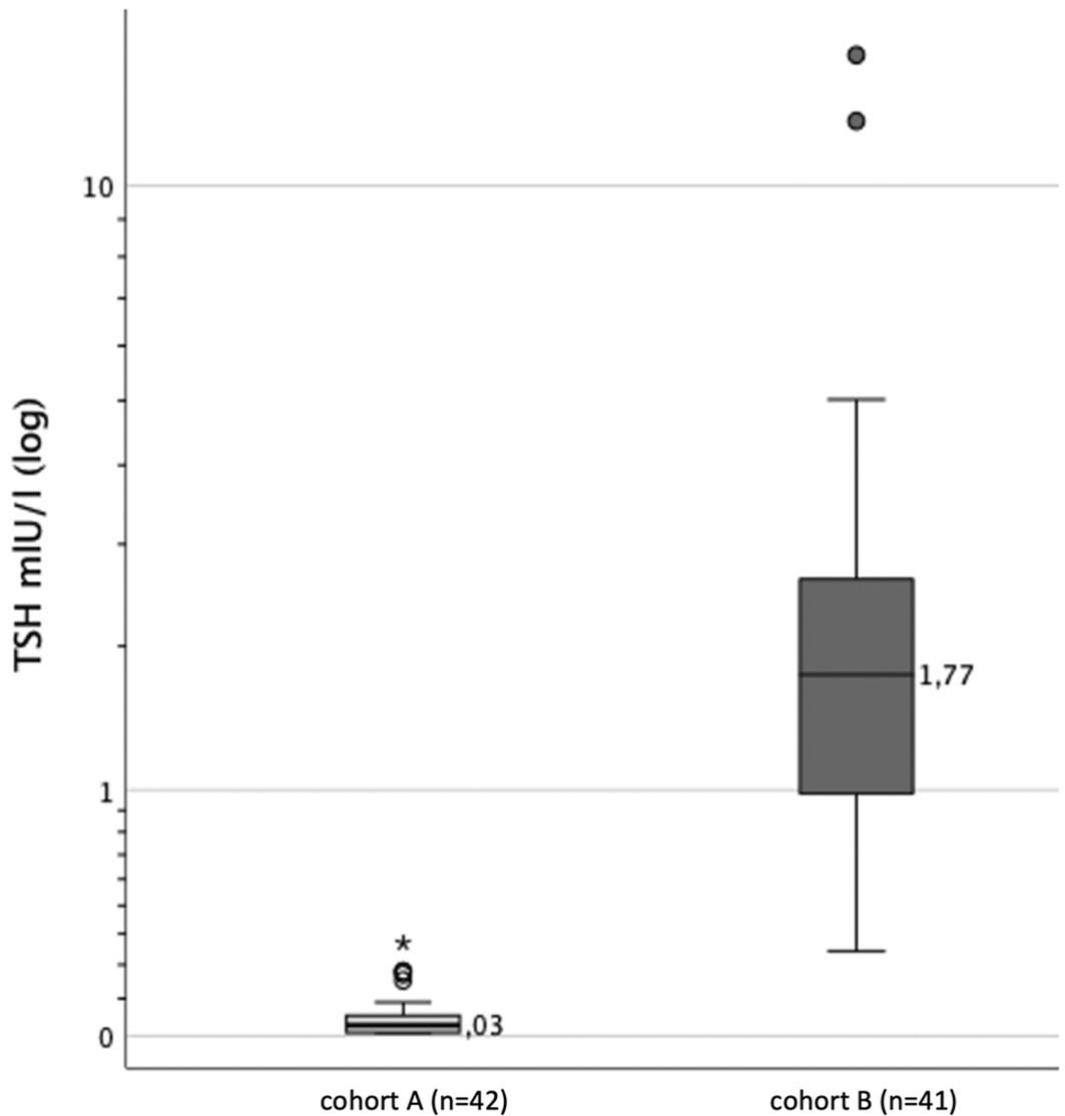
TSH distribution (with median) considering both cohorts (logarithmic scaling).

Proportion of female and male patients within both groups were equally distributed, *p* = 0.916 (Table 1). Moreover, a non-significant difference in age (z = -0.565, *p* = 0.572) (Table 2) and BMI (z = -0.829, *p* = 0.407) was revealed when comparing the two cohorts as well as a significant difference in TSH, indicating a large effect (z = -7.848, *p* < 0.001, r = 0.86) (Table 3). The distribution of TSH in both cohorts is illustrated in Fig. 1.

**Table 1:** Frequency and proportion (column percentage) of patient’s sex.

Sex	Cohorts		Total
	Cohort A	Cohort B	
Male	20 (47.6%)	20 (48.8%)	40 (48.2%)
Female	22 (52.4%)	21 (51.2%)	43 (51.8%)
Total	42 (100%)	41 (100%)	83 (100%)

**Table 2:** Characteristics of age (years) considering both cohorts.

	n	*M* ± *SD*	Min—Max	*Md*	IQR	Mean rank
Cohort A	42	62.2 ± 13.5	32.8—82.8	61.9	54.0; 73.8	43.48
Cohort B	41	61.5 ± 10.9	43.9—86.3	59.6	53.2; 65.6	40.49
Total	83	61.9 ± 12.2	32.8—86.3	59.6	53.4; 72.3

**Table 3:** Key values of TSH (μU/mL) considering both cohorts.

	n	*M* ± *SD*	Min—Max	*Md*	IQR	Mean rank
Cohort A	42	.055 ± .066	.009—.300	.030	.010; .063	21.52
Cohort B	41	2.40 ± 2.83	.270—14.90	1.77	.965; 2.64	62.98
Total	83	1.21 ± 2.30	.009—14.90	.270	.030; 1.77

As already mentioned above, only patients with at least six months of TSH suppression therapy were included in cohort A. Duration of therapy lasted 4.45 ± 2.64 (min 0.82—max 9.47; Md = 4.23, IQR 2.01—6.99) years.

**Figure 2:**
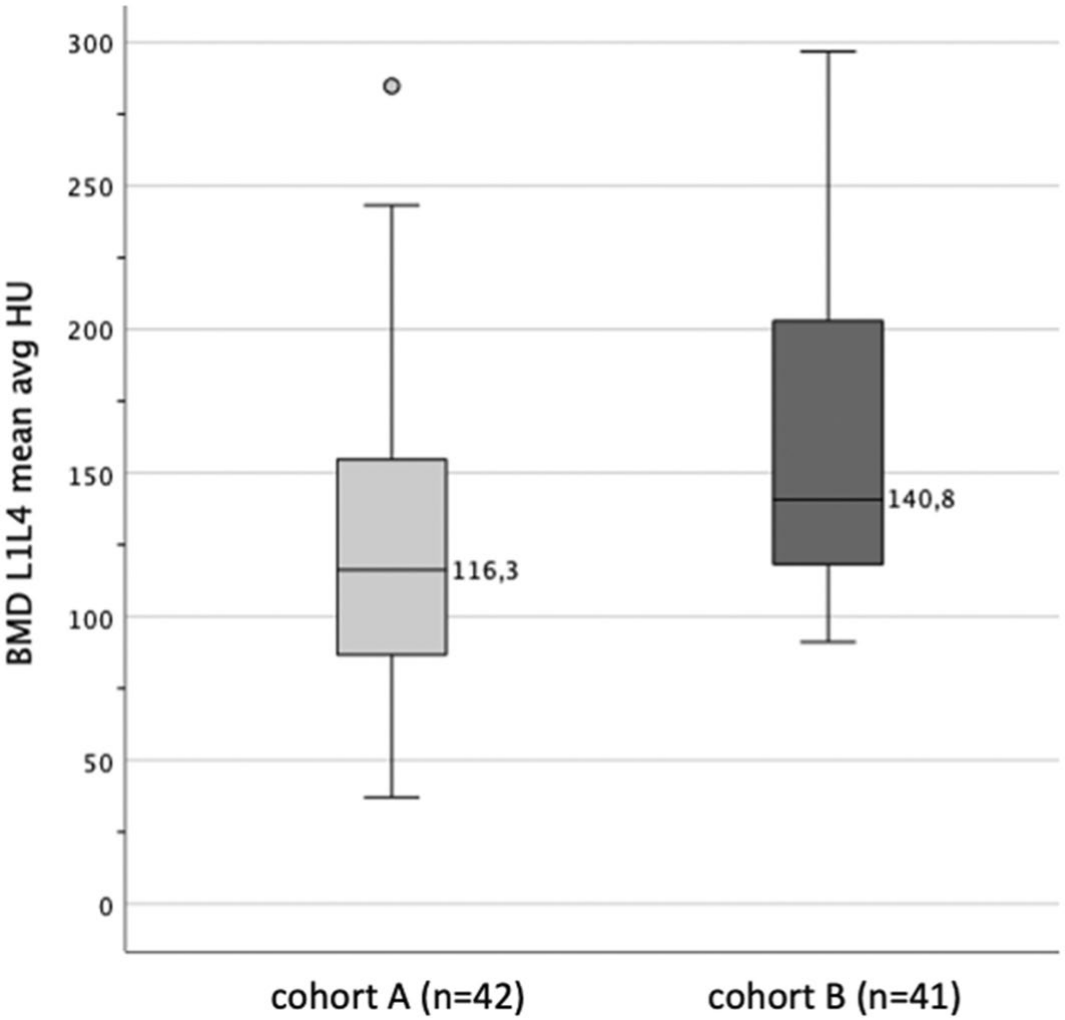
Distribution (with median) of BMD^HU m considering both cohorts.

BMD^HU m for L1- L4 was found to be significantly higher in cohort B with moderate effect (*p* = 0.006; *r* = 0.30). Figure 2 illustrates the distribution of BMD^HU for the two cohorts.

The result of the two-way ANOVA revealed no significant interaction of cohort * sex, *F* (1, 79) = 0.322, *p* = 0.572. Table 4 displays the characteristic values depending on the 2 × 2 ANOVA. No significant difference in BMD^HU was observed for sex, *F* (1, 79) = 0.168, *p* = 0.683. However, a significant difference with a moderate effect (η^2^ = 0.08) was observed for the factor cohort, *F* (1, 79) = 6.802, *p* = 0.011. This result indicates a higher BMD^HU (20.4%) for cohort B compared to cohort A. Comparing patients` sex separately, a significant difference of BMD^HU m was determined in men (*p* = 0.023), and tendentially in women (*p* = 0.094).

**Table 4:** Characteristics (n, M ± SD, Md (IQR)) of averaged BMD^HU considering sex and both cohorts.

	Male	Female	Total
Cohort A	20	22	42
	121.39 ± 39.69	133.77 ± 73.57	127.87 ± 59.51
	117.68 (98.10–151.25)	116.25 (81.75–197.0)	116.25 (86.78–154.75)
Cohort B	20	21	41
	161.65 ± 57.66	159.65 ± 53.14	160.63 ± 54.70
	143.88 (117.50–192.75)	135.75 (121.67–203.0)	140.75 (118.25–203.0)
Total	40	43	83
	141.52 ± 52.94	146.41 ± 64.98	144.05 ± 59.18
	131.13 (109.13–160.25)	129.0 (107.06–200.0)	129.0 (107.75–182.50)

Regarding the relation between age and BMD^HU in both cohorts and considering sex, a significant negative correlation was apparent, *r* = -0.62 (*p* < 0.001, two-tailed). This indicated that bone density decreased with increasing age. The correlations were slightly stronger for female patients (*r* = -0.72) than for males (*r* = -0.53).

In addition, MANOVA was performed to examine the differences between the two cohorts regarding the averaged BMD^HUs from the L1 to L4 vertebrae. It is important to mention that this analysis was performed only with fully protocolled measurements in each case (n = 75). The results indicate that patients in cohort A showed slightly lower BMD compared to those in cohort B. In particular, a significantly lower bone density could be found for L1 (*p* = 0.048) with a small effect (η^2^ = 0.05). The extents of the differences from the L2- L4 vertebrae can be interpreted as a trend (Table 5).

**Table 5:** Characteristics (M ± SD, Md (IQR)) of averaged BMD^HU L1-L4 considering both cohorts.

BMD^HU	Cohort A (n = 36)	Cohort B (n = 39)	Total (n = 75)	*p*-value	Effect η^2^
L1	139.28 ± 58.47	165.54 ± 54.84	152.93 ± 57.75	.048*	.05
	128.5 (101.7; 176.5)	145.0 (124.5; 192.0)	140.0 (117.0; 191.0)		
L2	135.66 ± 64.93	162.85 ± 56.18	149.80 ± 61.66	.056	.05
	126.5 (91.4; 162.0)	145.0 (117.5; 201.5)	140.0 (108.0; 191.0)		
L3	134.87 ± 60.50	159.48 ± 55.27	147.66 ± 58.76	.070	.04
	119.5 (88.2; 172.0)	136.0 (122.5; 190.0)	133.0 (109.0; 183.0)		
L4	131.38 ± 60.56	154.30 ± 58.20	143.30 ± 60.05	.099	.04
	116.5 (93.0; 171.5)	134.0 (112.5; 186.0)	132.0 (106.0; 182.0)		

*p ≤ .05.

No significant differences could be observed for vitamin D (p = 0.831, *r* = 0.03) and free T3 (*p* = 0.505, *r* = 0.08) comparing the two cohorts. However, results indicated that calcium and free T4 were significantly higher in cohort A, with small effects for calcium (*p* = 0.031, *r* = 0.24) and large effects for free T4 (*p* < 0.001, *r* = 0.68).

Furthermore, the averaged BMD^HU in the L1-L4 vertebrae of 17 patients within cohort A was examined for changes in the post-therapeutic period, lasting for 2.3 ± 1.2 (min 0.8—max 4.0; Md 1.9, IQR 1.1; 3.5) years.

**Figure 3:**
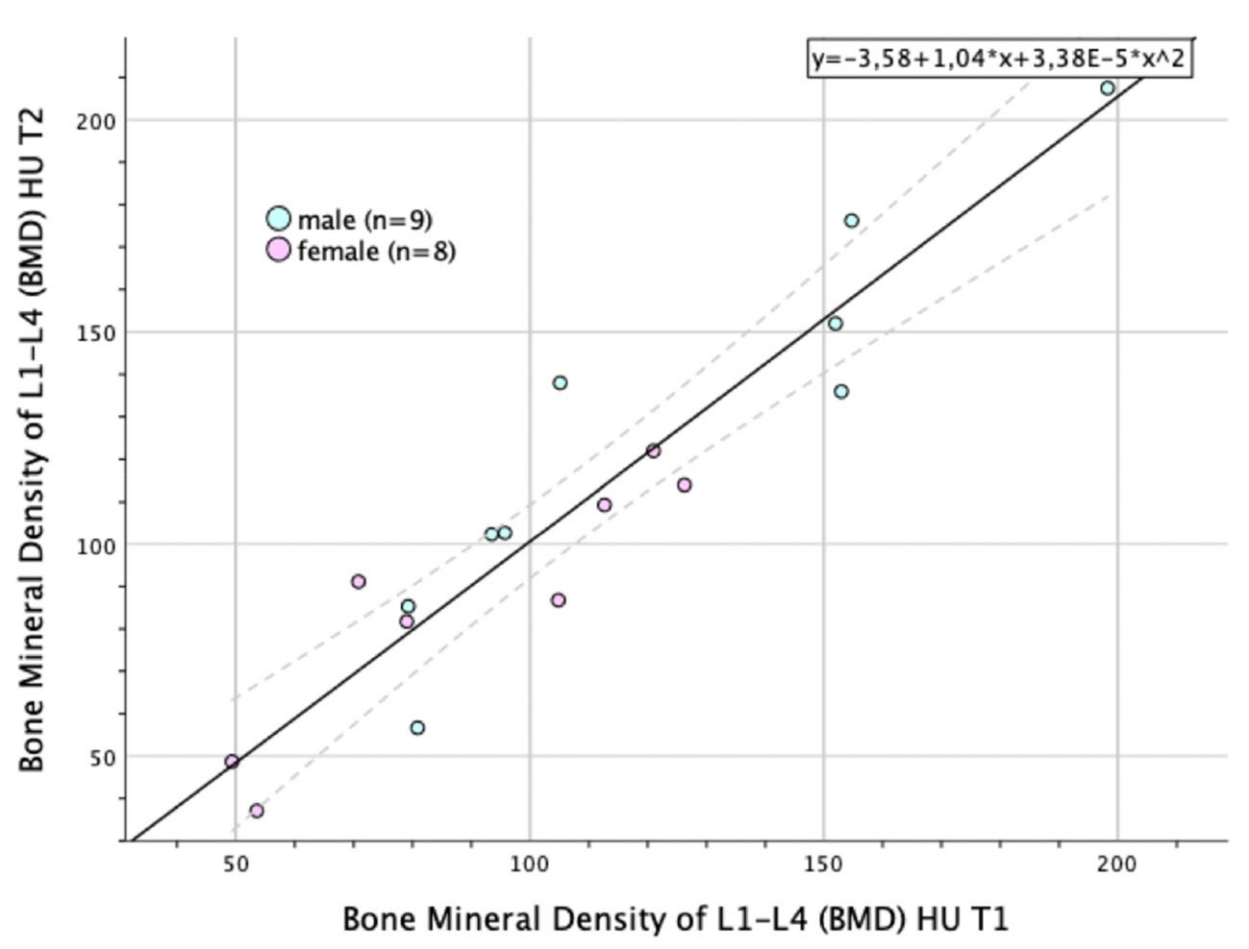
Bivariate scatterplot depicting change in BMD within the post-therapy time period (T1-T2), adjusting for patient sex with quadratic regression function and CI-95% (n = 17, cohort A).

No significant change in BMD^HU could be found between the two PET/CTs at different time points in the post-therapeutic period, T1 (107.65 ± 39.82) vs. T2 (108.69 ± 44.42), with *t* (16) = -0.276, *p* = 0.786. The Pearson's correlation coefficient of *r* (17) = 0.94 (*p* < 0.001) supports these results and indicated the substantial stability of BMD^HU within this cohort (Fig. 3).

## Discussion

Based on current knowledge, whether TSH suppressive therapy leads to decreased bone density is still controversial.

Ross et al. examined premenopausal women who were taking prescribed prolonged suppressive doses of L-thyroxine due to different diseases of the thyroid. A reduction of 9% in bone density could be found in those 12 women who had taken L-thyroxine for at least 10 years^30^. Nevertheless, it is also important to mention that the mean serum thyroxine concentration and free thyroxine index in this American study were slightly above the reference range. A study of patients suffering from thyroid cancer, by Diamond et al., included 24 women who underwent total thyroidectomy for non-anaplastic thyroid cancer, iodine ablation therapy, and subsequent suppressive therapy. They determined a significantly reduced femoral neck BMD in premenopausal and postmenopausal women, as well as a significantly reduced lumbar spine BMD in postmenopausal women^29^.

Those previously mentioned analyses especially focused on pre- and postmenopausal women. Interestingly, various studies have shown an age- and postmenopausal-related decrease of BMD, in general^40,41^. In women, this effect on BMD occurs predominantly after menopause and is mainly driven by estrogen deficiency^42^.

Other studies suggest opposite outcomes. Görres et al. compared BMD measured by dual-energy X-ray absorptiometry in 65 patients with DTC who were receiving thyroxine replacement therapy in suppressive doses to an age- and sex-matched control group. No significant difference between both groups could be found regarding BMD^33^. Another study, focused on a healthy middle-aged Korean population, examined the relationship between subclinical thyroid dysfunction and BMD. The BMD did not differ significantly in 146 patients with subclinical hyperthyroidism compared to patients with normal TSH levels^43^. However, it should be noted that this study was based on a population with only one single serum TSH measurement and no requirement for suppressive TSH levels.

In our study, there was a critical difference in bone densities in euthyroid patients with normal TSH values in cohort B compared to patients with prolonged suppressed TSH values (4.45 ± 2.64 years) assigned to cohort A (Fig. 1). Although both cohorts were comparable regarding potential confounders age and sex, cohort B showed a 20.4% higher BMD^HU than cohort A. Interestingly, especially men showed a significant difference (*p* = 0.023) in mean BMD^HU. Nevertheless, the extents of differences in women (*p* = 0.094) can be interpreted as a non-significant trend (Table 4).

It is important to point out that our study design is unique and differs from the others in its use of CT rather than DXA to predict BMD. Despite this fact, our results are in accordance with several other studies^29–31^, and lead to the assumption that long-lasting TSH suppressive therapy causes a decrease in BMD, and therefore, should be seen as a risk factor for osteoporosis. In addition, our patient population was divided equally between male and female patients, and thus, can be seen as valid for both genders.

Furthermore, we discovered that there was no significant difference regarding BMD^HU in those patients who received a second PET/CT scan after 2.3 ± 1.2 years. This result also correlates with a study from Gürlek et al., who evaluated 15 premenopausal women with endogenous subclinical hyperthyroidism for between six and 11 months, and who did not show reduced BMD^36^. A more recent study from Wang et al. observed 29 DTC patients 1–2 years postoperatively in whom there was no significant effect on BMD^32^. Most likely, these outcomes are related to the short time window between the two measurements. However, these results suggest that a long-lasting TSH suppression therapy is a prerequisite for a relevant reduction in BMD.

In the future, TSH suppression therapy should be evaluated in each patient depending on the individual risk of persistent or recurrent disease, as well as on the aggressiveness of the tumor. The possibility of complications due to this therapy must be weighed against the risk of increasing tumor cell proliferation.

Our results indicate that anti-osteoporotic prophylaxis should be considered during the duration of TSH suppression therapy to avoid the high risk of reduced bone density.

### Limitations

First, the measurement of the bone densities of the L1-L4 vertebral bodies cannot be considered exactly repeatable, as the manner in which it is performed can vary and could have led to slight differences.

Second, due to thyroid hormone replacement therapy, free T4 was significantly higher in cohort A, which could have had an impact on our results.

Third, although DXA is considered the gold standard for the measurement of BMD, HU measurements obtained from CT scans can predict BMD as well, according to various studies. Nevertheless, this could be seen as a limitation factor.

Additionally, due to the study design, we were not able to examine the trabecular bone score (TBS), which would have been additional information with which to predict fractures independently of BMD.

## Conclusion

In summary, according to our data, long-lasting TSH suppression therapy causes a statistically significant decrease in bone density, and therefore, a higher likelihood of osteoporosis in those patients.

In contrast, no statistically significant decrease in bone density was observed after short-term TSH suppression. This indicates that patients must be under prolonged TSH suppression to be at risk for a significant decrease in bone density.

## Data Availability

The datasets used and analysed during the current study is available from the corresponding author on reasonable request.
